# The Effect of 6 and 12 months Duodenal-Jejunal Bypass Liner Treatment on Obesity and Type 2 Diabetes: a Crossover Cohort Study

**DOI:** 10.1007/s11695-017-2997-7

**Published:** 2017-11-06

**Authors:** Selwyn van Rijn, Bark Betzel, Charlotte de Jonge, David P. J. van Dijk, Ignace M. Janssen, Frits J. Berends, Nicole D. Bouvy, Jan Willem M. Greve

**Affiliations:** 1 Department of General Surgery, Maastricht University Medical Center, Maastricht, The Netherlands; 2 Department of General Surgery, Rijnstate Medical Center, Arnhem, The Netherlands; 3 Department of General Surgery, Zuyderland Medical Center, Heerlen-Sittard, The Netherlands; 4 Henri Dunantstraat 5, 6419 PC Heerlen, The Netherlands

**Keywords:** Obesity, Abdominal surgery, Duodenal-jejunal bypass liner, Type 2 diabetes mellitus

## Abstract

**Objective:**

The aim of this research was to study the duodenal-jejunal bypass liner (DJBL) treatment for obesity and type 2 diabetes mellitus (T2DM) in patients after dietary treatment in a cross-over design.

**Background:**

DJBL treatment has been proven effective for treatment of obesity and T2DM. However, data on safety and efficacy of a 12-month DJBL treatment is limited.

**Methods:**

In 2014, our research group reported on a multicenter randomized clinical trial. Patients were randomized to DJBL or dietary treatment (control group). Twenty-eight patients crossed over after their dietary treatment and received up to 12 months of DJBL treatment. Patient visits were conducted at baseline, during DJBL treatment (1 week, 1–6, 9, 12 months) and 6 months after removal of the liner. Patients underwent a standard physical examination, blood sampling, assessment of adverse events, nutritional and diabetes counseling, and a standardized meal tolerance test.

**Results:**

Of the 28 patients included in this study, 24 patients completed 6 months of treatment. Eighteen patients were extended to 12 months of DJBL treatment; 13 patients completed this treatment period. After 6 months of DJBL treatment, a significant increase in excess weight loss (EWL) and decrease in weight, BMI, HbA1c, fasting glucose, cholesterol, HDL and LDL improved significantly. After 12 months of DJBL treatment, these parameters stabilized.

**Conclusions:**

The DJBL is an effective, minimally invasive treatment option. Even after successful treatment with dietary restrictions, the DJBL is still capable of significantly reducing weight and improving cardiovascular and type 2 diabetes mellitus parameters in obese patients.

## Introduction

Worldwide over 600 million people suffer from obesity (BMI ≥ 30 kg/m^2^) [1, 2]. The population of the USA accounts for 13% of this obese population, which is approximately one third of the country’s population [3]. Indicating the magnitude of this disease, also in Europe 15–30% of the adult population is affected [4].

The obese population is at high risk to develop chronic diseases, such as metabolic disorders (diabetes mellitus, fatty liver disease), cardiovascular diseases, cancer, and wear and tear of the musculoskeletal organ [5–8]. Furthermore, obesity is associated with increased mortality [9]. Surgery has been proven most successful in the treatment of obesity and its comorbidities [10–12]. However, current surgical techniques are still accompanied by perioperative and postoperative complications. Not all patients can benefit from these surgical approaches since only patients with a BMI ≥ 35 with comorbidities or a BMI of ≥ 40 without comorbidities are, according to the current guidelines, considered for surgical treatment [12–14]. Several endoluminal techniques have been developed with the intent to reduce perioperative and postoperative complications while maintaining treatment success. These techniques also provide an alternative treatment option for the growing overweight and obese population (BMI between 25 to 35 kg/m^2^).

The duodenal-jejunal bypass liner (DJBL) is a promising endoluminal device mimicking the Roux-en-Y gastric bypass. The liner consists of an impermeable sleeve that is open at both ends to allow food passage. The liner is temporarily fixed in the duodenal bulb and extends into the jejunum. This way the pancreatic secretions and bile will only mix with the food distal to the liner, hereby creating a functional bypass of the duodenum and the proximal jejunum. Several studies investigating the DJBL have shown the procedure to be safe and effective with good results regarding weight reduction, improvement of type 2 diabetes parameters, and a decrease in cardiovascular parameters [15–19].

However, most studies investigated a treatment duration of 3–6 months and data on safety and efficacy of 12 months duration of treatment with the DJBL is limited.

The aim of this study was to evaluate the safety and efficacy of 12 months treatment with the DJBL in a randomized crossover study design, making the patient their own control. We hypothesize that treatment for 12 months with DJBL is safe and will accomplish a decrease in body weight accompanied by an improvement in blood glucose levels and cardiovascular parameters.

## Methods

In 2014, our research group reported on a large cohort study of 77 patients that were included in a multicenter randomized clinical trial conducted in the Netherlands at the Maastricht University Medical Center, the Zuyderland Medical Center (formerly known as the Atrium Medical Center) Heerlen, and the Rijnstate hospital Arnhem. During this clinical trial, patients were randomized either to the DJBL treatment group or to the diet control group.

In short, inclusion criteria consisted of a BMI ranging from 30 to 50 kg/m^2^, type 2 diabetes for less than 10 years, and a glycated hemoglobin A_1c_ (HbA_1c_) level between 7.5%–10%. In addition, patients were only allowed to take metformin, sulfonylurea (SU) derivatives, and/or insulin with a maximum dose of 150 IU. Most important exclusion criteria consisted of: prior weight loss of more than 4.5 kg, use of weight loss medication, innate insulin production failure as indicated by low C-peptide levels, GI tract abnormalities or prior surgery of the GI tract that could affect device placement, bleeding disorders, connective tissue disorders, and severe liver or kidney disease [16].

At baseline, patients were assessed extensively. Before the procedure, patient demographics and medical history were evaluated, and a physical examination (including weight, BMI, and blood pressure) was performed. Further patient evaluation consisted of blood parameter outcomes (diabetes parameters and cardiovascular parameters) and a standardized 4-h meal tolerance test using a liquid meal. Throughout the study, a diet with a maximum of 1200 kcal for women and 1500 kcal for men was prescribed of which the first week consisted of a liquids only regimen. Additionally, a diabetes nurse under supervision of an endocrinologist carried out the management of medical treatment of T2DM.

A total of 38 patients were randomized to the DJBL treatment group and 39 patients to the diet control group. Treatment protocol consisted of 6 months of DJBL treatment or control treatment with subsequently 6 months of follow-up. After 12 months, patients that were randomized to the diet group were offered the DJBL treatment for 6 months with an optional extension to 12 months treatment. We here describe this cross-over group.

Patient visits were conducted at baseline and during DJBL treatment at 1 week, monthly during the first 6 months, and for the extended treatment also at 9 and 12 months. In addition, patients were followed until 6 months after removal of the device.

During these visits, patients underwent a standard physical examination (including weight, BMI, and blood pressure), blood sampling, assessment of adverse events, and nutritional and diabetes counseling. Furthermore, a standardized meal tolerance test using a liquid meal (Ensure Plus vanilla flavor, Abbott Laboratories, Abbott Park, IL; 333 mL, 500 kcal, 20.8 g of protein, 67.3 g of carbohydrates, and 16.4 g of fat) was conducted at baseline, 3, 6, and 12 months during the DJBL treatment.

The study was conducted according to the principles of the Standard ISO 14155: 2003 on clinical investigations with medical devices and the recommendations guiding physicians in biomedical research involving human patients adopted by the 18th World Medical Assembly, Helsinki, Finland, 1964 and later revisions and also in accordance with the guideline Medical Research Involving Human Subjects Act (WMO). The study was approved by the medical ethical committee of all three participating medical centers. The general principles of informed consent, ethics review, and data management were in line with good clinical practice (GCP).

### DJBL Procedure

The DJBL was developed to mimic the duodenal bypass component of the Roux-en-Y gastric bypass. The device consists of a 60-cm long impermeable fluoropolymer liner and a nitinol anchor, which is used to fixate the liner in the duodenal bulb. The procedure was performed endoscopically. After positioning of the endoscope in the stomach, the fluoropolymer liner was advanced into the duodenum and unfolded into the proximal jejunum. Removal of the DJBL was performed as previously described [15, 16].

### Statistical Analysis

All data are presented as median and interquartile ranges (IQR) since data were not normally distributed. Comparisons were performed using the Wilcoxon signed-rank test. The repeated measures Friedman analysis of variance was used to assess change over time. Post hoc testing was performed using the Wilcoxon signed-rank test with Bonferroni correction. *p* < 0.05 was regarded as significant. Missing data were not imputed. All statistical analyses were performed using commercially available computer software (IBM Corp. Released 2012. IBM SPSS Statistics for Windows, Version 21.0. Armonk, NY: IBM Corp.).

## Results

### Patient Characteristics

**Fig. 1:**
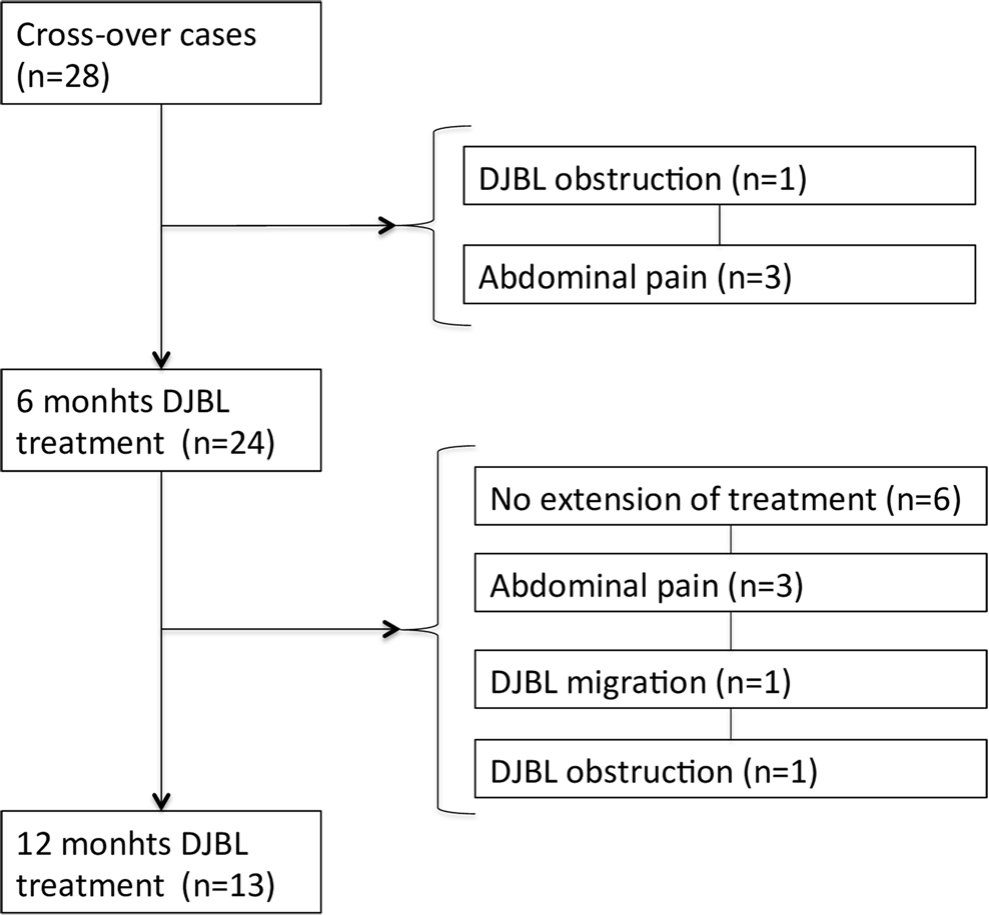
Flow-chart

Twenty-eight patients were implanted with the DJBL. Patient characteristics were compared between baseline and pre-implantation as described in Table 1 (17 males, median age 52 (48–56), 11 females, median age 48 (44–55)). Of these 28 patients, 18 were extended to 12 months of DJBL treatment. Reasons for early removal consisted of device related adverse events (Fig. 1). During the dietary treatment period, prior to 12 months implantation with the DJBL, patient characteristics already had significantly changed compared to baseline. This resulted in a significant difference prior to the DJBL intervention period for the following parameters: patients that were initially in the control group had a significant lower weight, BMI, systolic and diastolic blood pressure before implantation of the DJBL (Table 1).

**Table 1:** Comparison of weight, glucose metabolism and cardiovascular parameters between baseline and pre-implantation time point (*n* = 28)

	Baseline	6 months dietary treatment plus 6 months FU	*p* value
Sex (male/female)	17/11	–	–
Age (years)	50 (46–56)	–	–
Weight (kg)	113.4 (99.4–128.7)	110.4 (93.4–122.5)	0.001
BMI (kg/m^2^)	37.0 (33.0–42.9)	35.2 (31.7–40.0)	0.001
Duration of T2DM (years)	5.0 (3.3–7.8	–	–
HbA1C (%)	8.1 (7.7–8.8)	8.2 (7.0–8.8)	NS
Fasting glucose (mmol/L)	11.0 (9.1–8.8)	8.2 (7.0–8.8)	NS
Fasting insulin (pmol/L)	128.6 (83.4–284.5)	114.0 (57.7–165.8)	NS
Systolic blood pressure (mmHg)	152 (131–159)	137 (121–147)	0.001
Diastolic blood pressure (mmHg)	90 (80–95)	82 (77–90)	0.002
Total cholesterol (mmol/L)	4.3 (3.5–5.3)	4.4 (3.9–5.0)	NS
HDL (mmol/L)	1.2 (0.9–1.3)	1.2 (1.9–2.9)	NS
LDL (mmol/L)^1^	2.2 (1.7–2.8)	2.3 (1.9–2.9)	NS
Triglycerides (mmol/L)	1.8 (1.4–3.1)	1.7 (1.3–2.3)	NS

^1^ *n* = 25 patients included for analysis

### Effect of DJBL Treatment on Weight, Cardiovascular, and Type 2 Diabetes Parameters

**Fig. 2:**
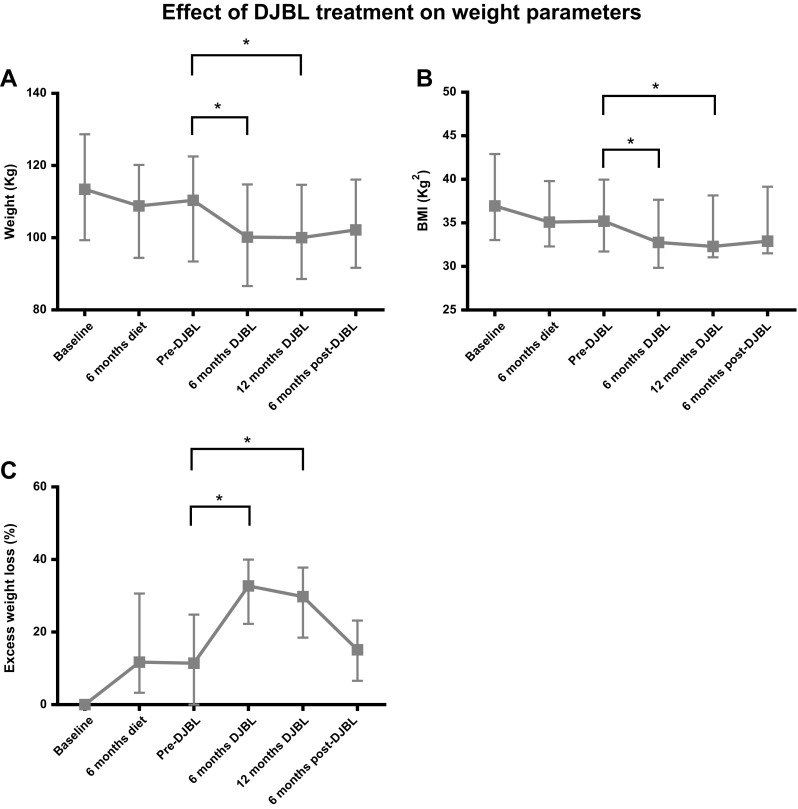
Effect of DJBL treatment on change in weight parameters over time. **a** Change in weight (kg). **b** Change in BMI (kg/m^2^). **c** Change in EWL (%). ^*^ *p* < 0.01

**Fig. 3:**
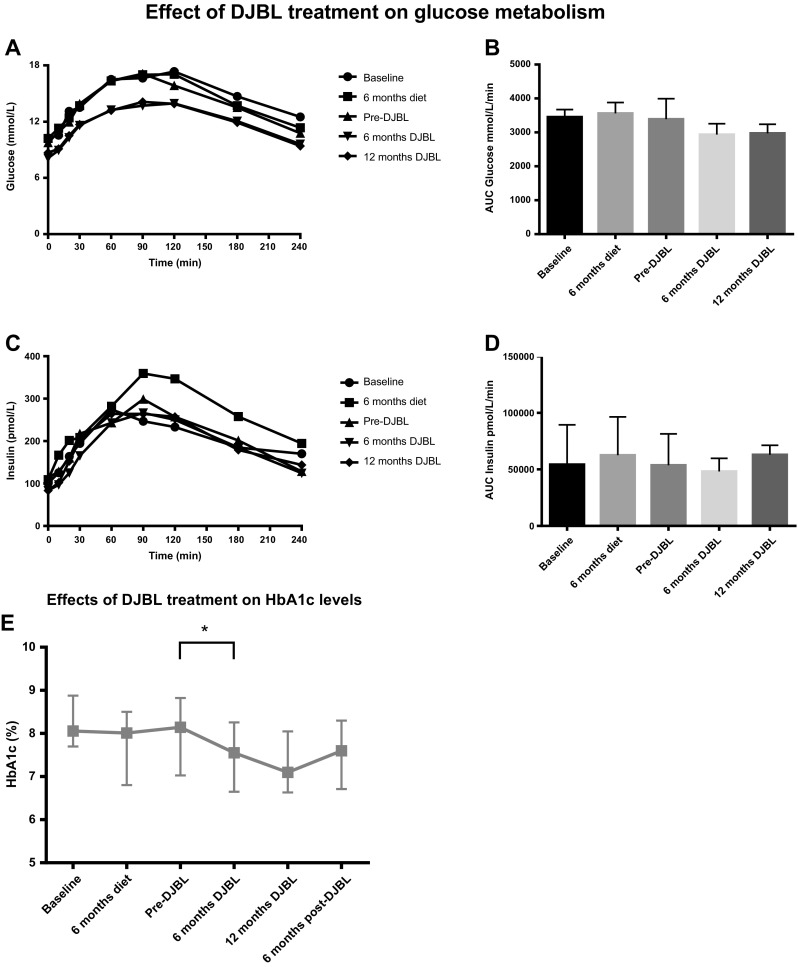
Effect of DJBL treatment on glucose metabolism parameters. **a** Glucose concentrations during the meal tolerance test. **b** The AUC calculations for glucose. **c** Insulin concentrations during the meal tolerance test. **d** The AUC concentrations for insulin. **e** Change in HbA1c (%). *p < 0.01

At 6 months of DJBL treatment, median weight was decreased from 109.7(92.2–122.5) kg to 100.2(86.7–114.8) kg (*p* < 0.001). Correspondingly, a median drop in BMI from 34.8(31.4–39.0) kg/m^2^ to 32.8(29.9–37.7) kg/m^2^ (*p* < 0.001) and an excess weight loss of 32.8(22.3–40.0) % (*p* < 0.001) was seen (Fig. 2). As shown in Fig. 3, HbA_1c_ decreased from 8.2(7.1–9.0) % to 7.6(6.7–8.3) % (*p* < 0.01). In addition, fasting glucose levels dropped from 9.7(8.8–13.3) mmol/L to 8.1 (7.1–9.8) mmol/L (*p* < 0.01). During 6 months of treatment, also a significant decrease in total cholesterol was seen from 4.5 (4.1–5.0) mmol/L to 3.9(3.2–4.4) mmol/L (*p* < 0.01). Simultaneously, HDL decreased from 1.2 (1.0–1.5) mmol/L to 1.1(0.78–1.25) mmol/L (*p* < 0.001) and LDL from 2.3(1.93–3.0) mmol/L to 2.1(1.5–2.4) mmol/L (*p* < 0.01). After 6 months of DJBL treatment, parameters remained stable during the extended treatment period up to 12 months.

### Overall Effect After 6 months Diet Plus 6 months Follow-up and DJBL Treatment

Weight parameters significantly decreased after diet followed by DJBL treatment compared to baseline. An overall change in weight of 10.3(7.1–16.4) kg (*p* < 0.001) with an EWL of 32.7(22.3–40.0) % (*p* < 0.001) and a drop in BMI by 3.7 points (2.5–5.0) kg/m^2^ (*p* < 0.001) was seen. Type II diabetes parameters also significantly changed with a decrease in HbA1c (0.8(−0.1–1.7) % (*p* < 0.05), fasting glucose (3.4(1.2–4.8) mmol/L (*p* < 0.001), and fasting insulin (69.2(27.8–190.4) pmol/L (*p* < 0.001). Cardiovascular parameters were also significantly reduced after dietary plus DJBL treatment. Total cholesterol decreased by 0.7 (0.2–1.5) mmol/L (*p* < 0.01). Additionally, triglycerides and HDL reduced by 0.4(−0.4–1.0) mmol/L (*p* < 0.05) and 0.1(0.0–0.29) mmol/L (*p* < 0.01), respectively. An overview of the results is listed in Table 2.

**Table 2:** Change in weight, glucose metabolism parameters, and cardiovascular parameters after 6 months of dietary treatment, 6 months of DJBL treatment, and dietary plus DJBL treatment

	6 months dietary treatment (*n* = 24)	6 months of DJBL treatment (*n* = 24)	Overall change after dietary and DJBL treatment
Weight loss (kg)	4.5 (1.1–8.6)	5.8 (2.6–11.2)	10.3 (7.1–16.4)
BMI loss (kg/m^2^)	1.5 (3.1–3.3)	2.0 (1.0–3.5)	3.7 (2.5–5.0)
Excess weight loss (%)	13.3 (2.1–28.4)	20.9 (10.0–28.8)	32.7 (22.3–40.0)
HbA1C (%)	0.6 (0.3–1.3)	0.5 (0.1–1.3)	0.8 (−0.1–1.7)
Fasting glucose (mmol/L)	1.7 (1.5–4.0)	1.6 (0.3–3.2)	3.4 (1.2–4.8)
Fasting insulin (pmol/L)^3^	23.3 (32.4–118.8)	27.1 (6.9–104.9)	69.2 (27.8–190.4)
Systolic blood pressure (mmHg)^1^	4 (3–26)	6 (4-12)	19 (5.5–28)
Diastolic blood pressure (mmHg)^1^	8 (4–11)	0 (2-5)	7 (−3–13)
Total cholesterol (mmol/L)	0.2 (0.2–0.4)	0.6 (0.1–1.1)	0.7 (0.2–1.5)
HDL (mmol/L)^1^	0.1 (0.0-0.2)	0.2 (0.1–0.3)	0.1 (0.0–0.29)
LDL (mmol/L)^2^	0.0 (0.2–0.3)	0.3 (0.0–0.8)	0.2 (−0.1–1.0)
Triglycerides (mmol/L)	0.1 (0.3–0.8)	0.2 (0.1–0.3)	0.4 (−0.4–1.0)

^1^ *n* = 23 patients included for analysis

^2^ *n* = 22 patients included for analysis

^3^ *n* = 18 patients included for analysis

### Six Months After Explantation of the DJBL

After 12 months of DJBL treatment, ten patients were eligible for 6 months of follow-up after explantation of the DJBL. Six months after removal of the DJBL weight and BMI increased from 99.5(86.1–114.2) kg to 102.8(90.2–116.0) kg and from 31.4(30.4–26.4) kg/m^2^ to 32.5(31.2–37.2) kg/m^2^ (both *p* = 0.02), respectively. Also, fasting insulin levels rose from 92.2(58.2–137.5) pmol/L to 203.5(99.9–415.5) pmol/L (*p* = 0.03). Regarding the other parameters, no significant increase was seen 6 months after DJBL explantation.

### Glucose-lowering Medication

During the study period, usage of glucose-lowering medication was assessed. Medication usage was classified as “increased” if the dose of one or more agents was increased or an additional glucose-lowering agent was added. Medication was classified as “decreased” if the dose of one or more agents was lowered, or if one or more agents were discontinued. All patients used glucose-lowering medication prior to implantation with the DJBL. Twenty-seven (96%) patients were taking metformin, 19 (68%) patients SU derivatives, and 13 (46%) patients were on insulin treatment. Alteration in glucose-lowering medication was evaluated in each of the 28 patients just prior to explantation of the DJBL. Glucose-lowering medication was decreased in 15 (54%) patients, increased in 8 (28%) patients and remained unchanged in 5 (18%) patients. Additional information on the use of metformin, SU derivatives, and insulin is described in Table 3.

**Table 3:** Change in glucose-lowering medication

	Change in glucose lowering medication postexplantation
Metformin	
Decreased, discontinued	14.8
Increased	18.5
SU derivatives	
Decreased, discontinued	44.4
Increased	22.2
Insulin	
Decreased, discontinued	40.0
Increased	40.0

Values are given in percentages

### Safety

During DJBL treatment, adverse events consisted mainly of minor gastrointestinal complaints (78.6%). Abdominal pain and discomfort was present in 39.3% of patients and nausea and vomiting were present in 31.1 and 14.3% of the patients, respectively. In addition, hypoglycemia occurred in 17.9% of patients. Complaints occurred primarily during the first 2 weeks of DJBL treatment after which most resolved.

During DJBL treatment, six adverse events required hospitalization. Four of these adverse events were device related, making early removal necessary. One patient was admitted because of complaints of upper abdominal pain due to an eversion of the liner after 1 month of treatment causing obstruction of the DJBL. A second patient was admitted because of abdominal pain and nausea caused by a food bolus blocking the liner after 9 months of treatment. Another patient developed postprocedure vomiting due to pylorospasm and in one patient a migration of the liner occurred after 9 months. After removal, all complaints resolved without sequelae. Additionally, during the study period, five patients withdrew because of ongoing abdominal pain, nausea, and vomiting. In summary, four patients (14%) had an early removal of the device. These patients were explanted before the 3-month treatment time point. In addition, five patients did not complete their extended period of DJBL treatment and were explanted between 9 and 11 months. No procedure related serious adverse events occurred.

## Discussion

In this study, we evaluated the impact of 6 and 12 months of DJBL treatment in a crossover cohort from a multicenter randomized clinical trial. This is the first study assessing the effect of DJBL treatment in a cohort of patients who first underwent dietary treatment prior to implantation of the DJBL.

In this patient group, 6 months of treatment with the DJBL showed a significant decrease in weight, and improvement of cardiovascular parameters and type 2 diabetes mellitus parameters. An extension to 12 months of treatment with the DJBL showed a stabilization of these parameters. In addition, in patients who underwent 12 months of DJBL treatment, 6 months after removal of the device only a significant increase for weight parameters was seen.

Overall results after dietary and DJBL treatment showed an even more pronounced decrease for weight, type 2 diabetes mellitus and cardiovascular parameters than expected. Our results indicate that 6 and 12 months of DJLB treatment can even lead to a further reduction in weight, cardiovascular, and diabetes parameters after a controlled diet period.

As mentioned earlier, the effect of the DJBL is attributable to its barrier function. Pancreatic and bile secretions will only mix with food distal to the liner, creating a functional bypass of the duodenum and the proximal jejunum. As a result, local absorption of micronutrients is decreased. Furthermore, it was recently shown that weight loss and improvement of obesity related comorbidities is accompanied by changes in satiety hormones. Postprandial GLP-1 and peptide YY increased during 6 months of DJBL treatment. At the same time, CCK and leptin concentrations decreased [20, 21]. It is known that these hormones play an important role in satiety experienced by both healthy and obese individuals [22, 23]. The changes in these incretins and leptin caused by implantation of the DJBL might explain the positive effects of this treatment.

Six months of treatment with the DJBL resulted in a significant excess weight loss and a decrease in weight, BMI, HbA_1c_, fasting glucose levels, total cholesterol, HDL, and LDL. This is in line with several other studies evaluating the effect of the DJBL that have also shown a decrease in weight, cardiovascular, and type 2 diabetes mellitus parameters [15–19, 24–27]. The previously published randomized clinical trial showed that DJBL treatment was superior in the reduction of these parameters compared to dietary treatment [16]. In this crossover study, as shown in Table 1, prior to DJBL implantation patients already had a significant decrease in weight, BMI, systolic and diastolic blood pressure. Our results show that the patients in the diet group performed exceptionally well. This might be explained by the intrinsic motivation of the patients to perform well during the diet period, since this was followed by implantation of the DJBL. This study demonstrates that after this controlled dietary treatment period, the DJLB is still capable of significantly reducing weight and improving cardiovascular and type 2 diabetes mellitus parameters.

After 12 months of DJBL treatment, patients seemed to reach a plateau phase. No difference was seen between 6 and 12 months of treatment with the DJBL. This is in accordance with recent literature, reporting that ongoing weight loss is diminished after approximately 6 months of DJBL treatment [28].

In this study, six serious adverse events were reported. Fourteen percent (4/28) required hospital admission because of device related adverse events. Additionally, almost all patients experienced minor adverse events. A recent study on safety experience with the DJBL showed a serious adverse event rate of 10%. In this study, adverse events consisted of GI bleeding, hepatic abscess, pancreatitis, and perforation of the anchor. Therefore, patients need to be well instructed and have close contact with a specialized DJBL treatment center because early and quick removal might be necessary in case of device and procedure related serious adverse events [29]. In the current patient population none of these serious complications occurred.

Some limitations to this study should be addressed. The design of the study is non-randomized. In addition, the number of patients is limited; it consists of 28 patients of which only 13 extended and completed their 12 months of treatment with the DJBL. The strength consists of patients functioning as their own control in this crossover designed study. This is the first study describing the effect of DJBL treatment after a controlled diet period.

In conclusion, the DJBL is an effective minimally invasive treatment option with the capability to reduce weight and improve cardiovascular and type 2 diabetes mellitus parameters in obese patients. Even after a significant reduction with dietary restrictions the DJBL is still capable of reducing these parameters even further.
